# Polymer-coated superparamagnetic iron oxide nanoparticles as T_2_ contrast agent for MRI and their uptake in liver

**DOI:** 10.4155/fsoa-2017-0054

**Published:** 2017-09-18

**Authors:** Lamiaa MA Ali, Pasquina Marzola, Elena Nicolato, Silvia Fiorini, Marcelo de las Heras Guillamón, Rafael Piñol, Lierni Gabilondo, Angel Millán, Fernando Palacio

**Affiliations:** 1 Instituto de Ciencia de Materiales de Aragón, CSIC – Universidad de Zaragoza; & Departamento de Física de la Materia Condensada, Facultad de Ciencias, 50009 Zaragoza, Spain; 2 Department of Computer Science, Verona University, Verona, Italy; 3 Department of Neurological & Movement Sciences, Verona University, Verona, Italy; 4 Department of Animal Pathology, Unit of Histology & Anatomical Pathology, Zaragoza University, 50013 Zaragoza, Spain

**Keywords:** contrast agent, Endorem^®^, liver, magnetic resonance imaging, MRI, SPIONs, superparamagnetic iron oxide nanoparticles, toxicity, uptake

## Abstract

**Aim:**

To study the efficiency of multifunctional polymer-based superparamagnetic iron oxide nanoparticles (bioferrofluids) as a T_2_ magnetic resonance contrast agent and their uptake and toxicity in liver.

**Materials & methods:**

Mice were intravenously injected with bioferrofluids and Endorem^®^. The magnetic resonance efficiency, uptake and *in vivo* toxicity were investigated by means of magnetic resonance imaging (MRI) and histological techniques.

**Results:**

Bioferrofluids are a good T_2_ contrast agent with a higher r_2_/r_1_ ratio than Endorem. Bioferrofluids have a shorter blood circulation time and persist in liver for longer time period compared with Endorem. Both bioferrofluids and Endorem do not generate any noticeable histological lesions in liver over a period of 60 days post-injection.

**Conclusion:**

Our bioferrofluids are powerful diagnostic tool without any observed toxicity over a period of 60 days post-injection.

## Lay abstract

Several superparamagnetic iron oxide nanoparticles (SPIONs) preparations have been approved by US FDA for clinical use as MRI contrast agents. In recent years, we have been developing a synthetic multifunctional platform for SPIONs based on the use of polymers. In this report, we explored the diagnostic potential of these nanoparticles (herein called bioferrofluids) as an MRI contrast agent and their uptake in liver, without neglecting their toxicological effects. Results show that our bioferrofluids are a good T_2_ contrast agent without any observed toxicity in liver.

## Introduction

Over the past few decades biological applications of nanomaterials have become a subject of intense research activity [1]. This has been particularly so since around the beginning of this century in the case of superparamagnetic iron oxide nanoparticles (SPIONs) [2]. Particles of about 20 nm or less have unique properties that adjust very well to functionalities of interest in biomedical applications. Besides their size being comparable to many biological objects, their magnetic behavior is superparamagnetic, so they can be easily magnetized in the presence of an external magnetic field, their magnetization coming to zero as soon as the field is suppressed [3]. Their magnetization values are orders of higher magnitude compared with the values corresponding to transition metal or lanthanide ions, so they can strongly affect the spin–spin relaxation times (T_2_) of nearby water protons. This makes magnetic nanoparticles (MNPs) excellent contrast agents (CAs) in MRI [3–5]. Their strong magnetization also allows them to be fixed, moved, tagged or detected magnetically thus making them useful in bioseparation, diagnosis or in targeted drug delivery [6,7]. Moreover, they can also respond resonantly to an alternating magnetic field and induce heating, therefore being of interest for magnetic-induced hyperthermia [8–10].

MRI is considered one of the most promising non-invasive diagnostic tool in medical science since it provides 3D anatomical images with high spatial resolution in the submillimeter range and high soft tissue contrast [11]. Several SPION preparations have been approved by US FDA for clinical use as magnetic resonance (MR) CAs, such as Endorem^®^ for liver imaging [12].

The successful biomedical application of a nanomaterial is a reflection of its adequate design. Several factors should be taken into account such as size [13–15], shape [16], surface charge [17] and surface modification [18], to list but a few. Regarding surface modification (e.g., coating [13]), coating the nanomaterial with a polymeric coating reduces protein adsorption to nanomaterial surface and subsequently can reduce the nanomaterial clearance from the circulation through opsonization process. The most commonly used polymer is polyethylene glycol (PEG), since it is inexpensive, versatile and is currently listed as ‘generally recognized as safe’ by the FDA. Addition of PEG to the nanomaterial surface (PEGylation) increases the blood circulation time and reduces the non-specific binding of proteins, depending on the chain length of PEG. Chain lengths of about 2 kDa molecular weight are considered very suitable for this purpose, although much longer chains have also been proposed [19–22].

In the recent years we have been developing a synthetic multifunctional platform for SPIONs based on the use of polymers [23,24]. They consist of:

- Maghemite (*γ*-Fe_2_O_3_) cores that are embedded in
- A hydrophobic, poly(4-vinyl pyridine) (P4VP) matrix, covered by
- A shell of a second hydrophilic polymer, PEG, that confers to the material high solubility, stability in aqueous solutions and biocompatibility, and
- Other physical functionalities such as fluorescent dyes.

**Figure 1.**
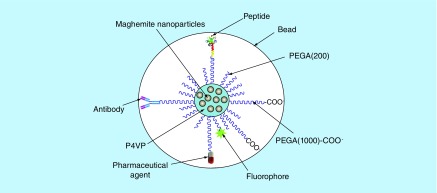
**Schematic representation of multicore magnetic nanoparticles with their polymeric multifunctional coating.**

Part of the PEG chains are functionalized with –COOH groups to provide sites for the anchoring of antibodies or other biological substances to the nanoparticle surface, so they can be directed to specific targets. The nanoparticle core consists of a small number of maghemite MNPs, for which diameters can be varied from 4 to 25 nm, all embedded within the P4VP matrix, thus preventing agglomeration, and then coated with PEG. The final hydrodynamic diameter of the nanoparticle including core and polymeric coatings can be varied from 50 to 160 nm. Altogether, the system consists of multicore nanobeads (Figure 1) that can be dispersed in phosphate-buffered saline (PBS), thus resulting in a magnetic fluid stable at pH = 7.4 and human body temperature that in the following will be designated as bioferrofluid [24].

*In vitro* cytotoxicity studies of these nanoparticles showed little incidence of oxidative stress, and inflammasome activation was only observed with the smaller nanoparticles at high concentration [25].

The hemostatic behavior of these bioferrofluids indicates no significant change in the complete blood counts and absence of hemolysis. Coagulation studies indicate that these bioferrofluids behave as non-specific anticoagulant, as lengthening of the activated partial thromboplastin time was detected, the degree of activated partial thromboplastin time lengthening being comparable to that of heparin and well within therapeutic range [26].

Stable colloidal suspensions of these nanoparticles have shown excellent relaxometric and magnetothermic responses compared with Endorem [27,10].

Altogether these studies make these bioferrofluids as good candidates for theranostics.

The purpose of this work is to investigate the MR efficiency of such polymeric-coated multicore nanoparticles as a T_2_ MR CA and their uptake and toxicity in mice using MRI and histological techniques. Studies concentrate on liver; uptake and toxicity effects in other organs are in preparation and will be published elsewhere. The results will be contrasted with those obtained using commercialized Endorem magnetic fluids following the same experimental conditions.

## Materials & methods

### Bioferrofluids preparation & characterization

The synthesis of bioferrofluids was performed using co-precipitation method through a polymeric route. The synthesis was carried out in two steps: synthesis of maghemite/P4VP nanocomposite and formation of bioferrofluids by dispersion of the nanocomposite in acidic pH and coating with a hydrophilic polymer (PEG acrylate [PEGA]). Two types of PEGA chains were used, one of them with 200 Da molecular weight [PEGA(200)] and hydroxyl terminal groups, and the other with 1000 Da molecular weight and carboxyl terminal groups [PEGA(1000)-COO] that can be used for further functionalization. The polymers were added in a 9:1 ratio per weight, respectively, and were allowed to react with the P4VP by Michael addition reaction. The pH of the suspension was adjusted to 7.4 and the ionic strength to 0.15 M. The prepared bioferrofluids were finally filter-sterilized using a 0.22 μm nitrocellulose filter.

The total iron content in the samples was determined by atomic emission in a plasma 40 inductively coupled plasma Perkin–Elmer spectrometer. The size of the maghemite nanoparticles was determined from transmission electron microscopy (TEM) images while the hydrodynamic size distribution of the P4VP-g-PEGA-coated nanoparticles was determined by dynamic light scattering (DLS) using a Zetasizer Nano ZS from Malvern (Worcestershire, UK).

A detailed description of bioferrofluids preparation and characterization is provided in previous publications [24,26].

### *In vitro* relaxation measurement

*In vitro* MR imaging was performed with a Biospec Tomograph system (Bruker Medical systems, Karlsruhe, Germany) operating at 200 MHz (4.7 T) and equipped with a 33-cm bore magnet (Oxford Ltd, UK). The system was operating using Paravision 5.1 software (Bruker, Karlsruhe, Germany). Samples were prepared in physiological saline solutions at different concentrations of bioferrofluids and Endorem, then inserted in a 7.2 cm internal diameter (i.d) birdcage coil. Transverse (T_2_) and longitudinal (T_1_) relaxation times were measured. Measurement parameters are provided in the supplementary material section.

Longitudinal and transversal relaxation rates (1/T_1_, s^-1^ and 1/T_2_, s^-1^, respectively) were plotted as a function of iron concentration, expressed in mM of iron, and longitudinal (r_1_) and transverse (r_2_) relaxivities were obtained by the slope of the fitting straight line.

### *In vivo* perfusion MRI

A total number of five, 6-7 weeks old, male BALB/c mice weighting 19-22 g were used. Mice were purchased from Harlan Laboratories (Udine, Italy) and housed with free access to water and food, under controlled environmental parameters and veterinary control in the animal facility of the University of Verona.

Mice were pre-anesthetized and intravenously injected via the tail vein with aqueous suspensions of either bioferrofluid or Endorem^®^ at a dose of 23.5 mg Fe/kg body weight.

First-passage images were acquired using an echo-planar imaging sequence. Images were acquired during 60 s following the injection. The obtained signal intensity (SI) values were normalized according to the following equation:(1)
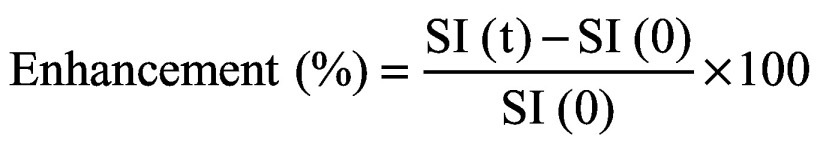



where SI (t) is the SI at time t, SI (0) is the SI before injection.

In order to evaluate the performance of the bioferrofluid as a CA compared with Endorem, experiments mapping the cerebral blood flow (CBF) and cerebral blood volume (CBV) bolus tracking (first passage) were performed [28]. Regional CBV (rCBV) can also be measured using blood pool CAs, at the steady-state concentration of CA in blood, by acquiring T_2_* images before and after CA injection and using the following relationship [29]:(2)
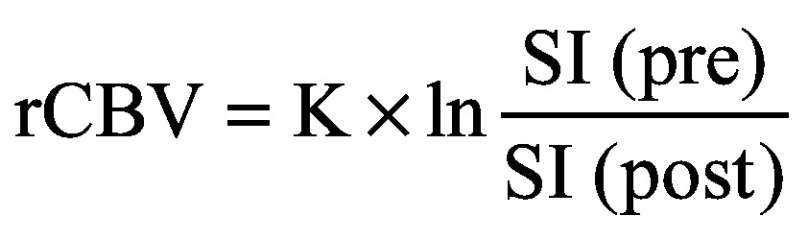



where, *K* is a constant depending on instrumental parameters; SI (pre) and SI (post) are brain SI values before and after CA injection, respectively.

Steady-state images were acquired before and during 2 h after injection of CA using a gradient echo sequence.

A detailed description of the methodology with the measurement parameters are provided in the supplementary material section.

### Liver MRI study

Mice were intravenously injected via the tail vein with aqueous suspensions of either bioferrofluids or Endorem at a dose of 20 μmol Fe/kg body weight. Images for the whole body were taken before injection (pre) and at different time points after injection (5 min–2 h, 24 h and 7, 15, 30 and 60 days). Quantitative T_2_ maps and T_2_*-weighted images were acquired using the parameters indicated in the supplementary material section.

### *In vivo* toxicity & qualitative iron detection studies

At each time point (2 h, 24 h and 7, 15, 30 and 60 days), animals were sacrificed through cervical dislocation; dissected tissues were processed for toxicity studies and qualitative iron detection using Prussian blue assay. Methodologies are described in details in the supplementary material section.

### Statistical analysis

Obtained data were presented in the curves as (mean ± SEM), the number of readings at each time point is indicated by symbol (n), data were analyzed using Mann–Whitney test. p < 0.05 was considered statistically significant.

## Results

### Bioferrofluids characterization

**Figure 2.**
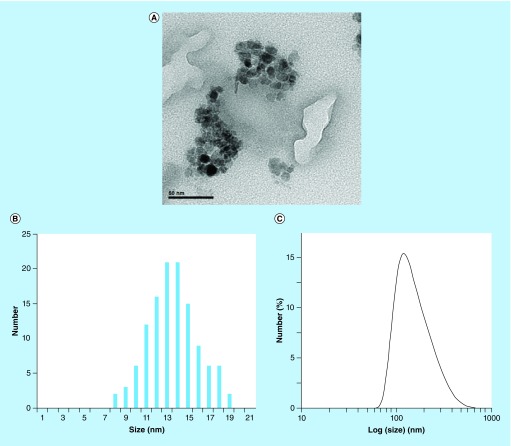
**Bioferrofluids characterization.** **(A)** TEM images of maghemite MNPs in the bioferrofluid; **(B)** size distribution of spherical maghemite MNPs; **(C)** DLS size distribution of hydrodynamic size in the bioferrofluids sample. DLS: Dynamic light scattering; MNPs: Magnetic nanoparticles; TEM: Transmission electron microscopy.

Bioferrofluids were characterized by TEM and DLS and the results are shown in Figure 2. TEM images show that the polymer coating did not give an appreciable contrast against the carbon film of the sample holder; thus, only the maghemite MNPs were visible. Figure 2A shows TEM images of several multicore nucleus with sizes of several tenths of nanometers. The individual maghemite MNPs in the nucleus are rounded and their average size from the analysis of TEM images was 13.08 ± 2.33 nm (Mean ± SD) (Figure 2B). DLS observations show a single population of nanoparticles with an average hydrodynamic diameter of 163 nm (Figure 2C). The Fe_2_O_3_ content of the bioferrofluids stock solution was 8.1 g/l.

A well-known MRI CA, Endorem, (Guerbet, Villepinte, France), was used in this work as a reference material. Endorem is composed of magnetite nanoparticles with a size range between 6 and 9 nm, coated with a polymeric dextran. The hydrodynamic diameter of Endorem is ranging between 80 and 150 nm. Measurement of the hydrodynamic diameter in our laboratory yields an average of hydrodynamic diameter of 110 nm. The Endorem concentration is 11.2 g/l Fe.

### *In vitro* relaxation measurement

**Figure 3.**
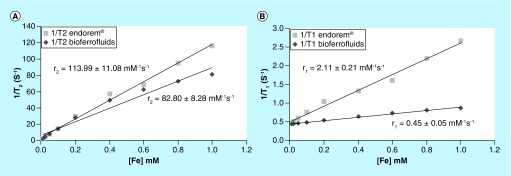
***In vitro* relaxation measurement.** Comparative analysis of the transverse relaxation rates (1/T_2_, s^-1^) **(A)** and longitudinal relaxation rates (1/T_1_, s^-1^) **(B)** of bioferrofluids and Endorem^®^ as a function of iron concentration (mM). r_2_ and r_1_ were calculated from the slopes of each plot.

T_2_ and T_1_ relaxation times were measured for both, Endorem and bioferrofluids. It was evident that these bioferrofluids exhibit the typical property of SPIONs of shortening T_2_ relaxation time, as increasing the nanoparticles concentration is associated with a decrease in SI, as shown in Supplementary Figure 1. Obtained values of transverse and longitudinal relaxation rates of bioferrofluids and Endorem have been plotted as a function of the iron concentration in mM in Figure 3. Both 1/T_2_ and 1/T_1_ relaxation rates are linearly proportional to the iron concentration as shown in Figure 3A & B. Relaxivity coefficient values (r_2_ and r_1_ ± C.I.) were obtained from the slope of the fitting straight lines. r_2_ values are 113.99 ± 11.08 and 82.8 ± 8.28 mM^-1^s^-1^, while r_1_ values are 2.11 ± 0.21 and 0.45 ± 0.05 mM^-1^s^-1^ for Endorem and bioferrofluids, respectively. It is worthwhile to mention that, at high iron concentration, the trend of 1/T_2_ for bioferrofluids slightly deviates from linearity and consequently r_2_ for bioferrofluids may be underestimated. Indeed, if we assume that this deviation can be attributed to experimental artifacts and eliminate the last two points, the calculated transversal relaxivity for bioferrofluids amounts to 104.7 ± 9.7 mM^-1^s^-1^, not statistically different from values reported for Endorem in the present and in other studies [30]. These results indicate that bioferrofluids have transversal relaxivity lower than Endorem or, at most, comparable. Transversal to longitudinal relativity values (r_2_/r_1_ ratios) were 54.02 and 184 for Endorem and bioferrofluids, respectively.

### *In vivo* perfusion MRI

**Figure 4.**
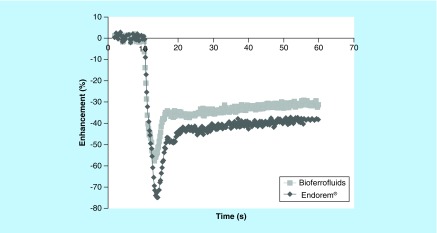
**A dynamic first-passage bolus tracking curve for both Endorem^®^ and bioferrofluids during an acquisition time of 60 s.**

A dynamic first-passage bolus tracking method was used in order to assess the usefulness of investigated CA in cerebral perfusion experiments. A first-passage CA curve, plotting the enhancement (%) versus the time point after injection is shown in Figure 4. These results clarify the effect of both Endorem and bioferrofluids on SI *in vivo*. Both cause a decrease in SI values with a different degree. SI decreases more with Endorem than with the bioferrofluids.

**Figure 5.**
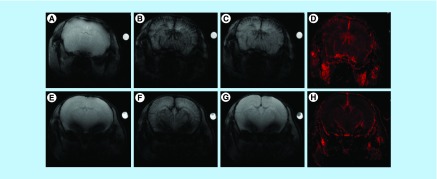
***In vivo* perfusion MRI.** T_2_*-weighted images for a mouse brain vasculature before **(A, E)**, 5 min **(B, F)** and 2 h **(C, G)** after injection of Endorem^®^ (upper panel) and bioferrofluids (lower panel). rCBV maps for Endorem and bioferrofluids are shown in **(D)** and **(H)**, respectively. rCBV: Regional cerebral blood volume

To evaluate the performance of the bioferrofluid as a CA compared with Endorem in steady-state condition, T_2_*-weighted images in brain were acquired before (pre) and during 2 h after (post) injection of Endorem and bioferrofluids at a dose of 23.5 mg Fe/kg body weight. Before injection, the brain vasculature appeared clear (Figure 5A & E). Few minutes after injection of Endorem and bioferrofluids, a decrease in SI was observed for both CAs (Figure 5B & F), which is indicated by a darkening in the brain vasculature (hypointense) emphasizing the existence of the CAs in the circulation. Two hours after injection, a hypointense brain vasculature was observed for the mouse injected with Endorem (Figure 5C); however, a cleared brain vasculature was observed for the mouse injected with bioferrofluids that emphasizes the clearance of bioferrofluids from the circulation (Figure 5G). Figure 5D & H shows rCBV maps calculated pixel-by-pixel according to Equation (2) with Endorem and bioferrofluids, respectively.

For a quantitative data analysis, SI values in T_2_*-weighted images were obtained by identifying the region of interest (ROIs) in the brain. These values were normalized to SI of the standard, and then the SI ratio (R) was calculated according to the following equation:(3)
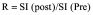



where, SI (post) is the SI after injection and SI (pre) is the SI before injection.

These data are plotted in Supplementary Figure 2 as a function of the acquisition time. It is evident that both CAs attenuate SI inside the brain in few minutes after injection, since R values were 0.51 ± 0.11 and 0.67 ± 0.16 (mean ± SD) compared with pre-injection values 1.00 ± 0.14 and 1.00 ± 0.18 for Endorem and bioferrofluids, respectively. With increasing time, a small increase in R was observed for Endorem. However, R observed for bioferrofluids shows a more pronounced increase, as is after 2 h it returns to the pre-injection value. Since CAs remain confined in the vascular space in normal brain, the drop in SI is attributable to iron contained in blood vessels, and consequently, the recovery of the parameter R to its pre-contrast value indicates, within the limit of the sensitivity of MRI, complete clearance of the bioferrofluids from the circulation. Supplementary Figure 2 indicates that bioferrofluids have cleared faster (i.e., have a shorter half-life time in blood) than Endorem.

### Liver MRI study

SI values in T_2_*-weighted images, as well as quantitative T_2_ values were obtained by identifying ROIs in liver. T_2_ values were obtained by fitting the theoretical dependence of SI on the echo time for the multi-echo acquisition sequence to experimental data.(4)
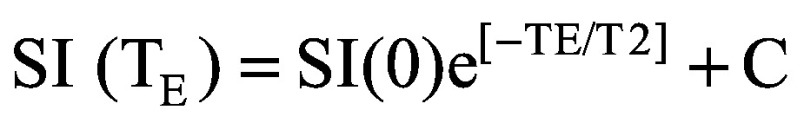



where, SI(T_E_) is the SI at certain echo time, SI(0) is the signal at zero time, T_E_ is the echo time, T_2_ is the transverse relaxation time and C is a constant. The fitting was performed pixel-by-pixel by using the image sequence analysis (ISA) tool of Paravision 5.1 software.

SI values in T_2_*-weighted images were normalized to SI of brain, which was not significantly different from the pre-injection value at the used dosage, and then R was calculated according to Equation (3).

**Figure 6.**
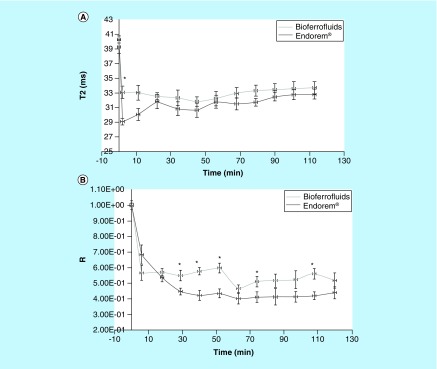
**Liver MRI study.** Transverse relaxation time values **(A)** and signal intensity ratio values **(B)** of bioferrofluids and Endorem^®^ in liver during 2 h after contrast agent injection. Data are presented as (mean ± SEM). *Marks significant differences between Endorem and bioferrofluids, according to Mann–Whitney test, n = 10.

Before injection, T_2_ values in the liver were 39.27 ± 2.98 (mean ± SD) and 40.30 ± 1.46 ms in the bioferrofluid and Endorem groups, respectively. Few minutes after CA injection, a decrease was detected in T_2_ values, 33.11 ± 2.93 and 29.11 ± 1.47 ms for bioferrofluids and Endorem injected mice groups, respectively, as represented in Figure 6A. These results are compatible with R results shown in Figure 6B, as a decrease in R values was also observed. The darkening in the liver tissue due to CA accumulation after 2 h of injection is shown in Supplementary Figure 3.

Estimation for the iron concentration in liver could be obtained from the following equation:(5)
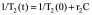



where, 1/T_2_(t) (in s^-1^) is the transverse relaxation rate of a system after CA administration, 1/T_2_(0) (in s^-1^) is the transverse relaxation rate of a system before CA administration, r_2_ (in mM^-1^S^-1^) is the relaxivity of the CA in the desired system and C is the concentration (in mM).

By using the experimentally determined T_2_ values for the liver in both bioferrofluids and Endorem after 2 h of injection and the r_2_ relaxivity (measured in physiological saline solution) we could estimate an iron concentration of about 0.05 μM in both cases.

**Figure 7.**
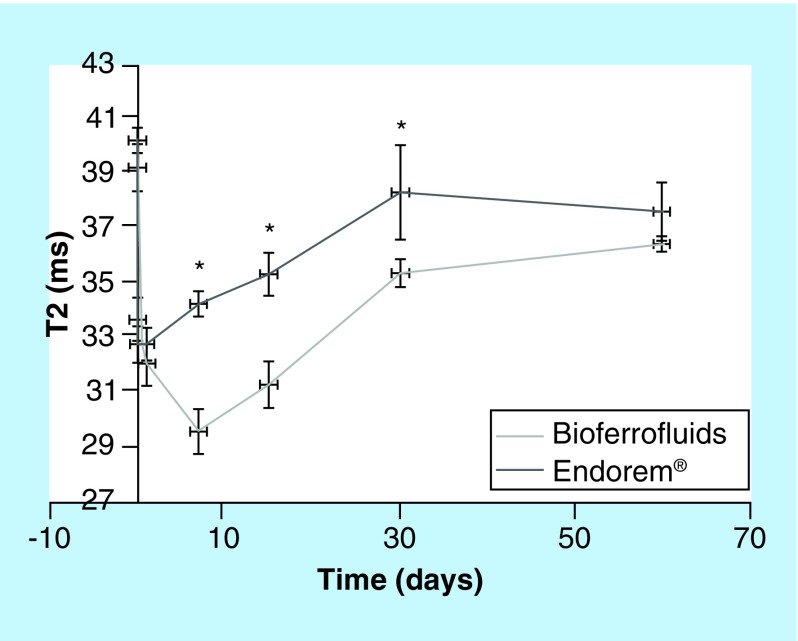
**Transverse relaxation time values of bioferrofluids and Endorem® in liver during 60 days after contrast agent injection.** Data are presented as (mean ± SEM). *Marks significant differences between Endorem and bioferrofluids, according to Mann–Whitney test, (until 15 days: n = 10; at 30 days: n = 8; and at 60 days: n = 4).

T_2_ and T_2_*-weighted images were also acquired at several time points after injection (24 h and 7, 15, 30 and 60 days) for liver. A decrease in darkness of the liver was evident with increasing time for both CAs indicating clearance from the liver Supplementary Figure 4. T_2_ values in liver have been calculated and plotted as a function of the acquisition time in Figure 7, data for R values are shown in Supplementary Figure 5.

After the sharp decrease in both T_2_ and R values in liver observed at 2 h after injection, increasing time was associated with an increase in T_2_ and R values which is expected as CA clearance from liver. Comparing the pre-contrast values to post-contrast values in both CAs, a significant difference is observed in T_2_ values between bioferrofluids and control (pre) until 30 days after injection, while in R there are significant differences only until 15 days after injection. In the case of Endorem, both T_2_ and R values show statistical significant differences to the control (pre) until 15 days after injection.

### Qualitative iron detection using Prussian blue assay

**Figure 8.**
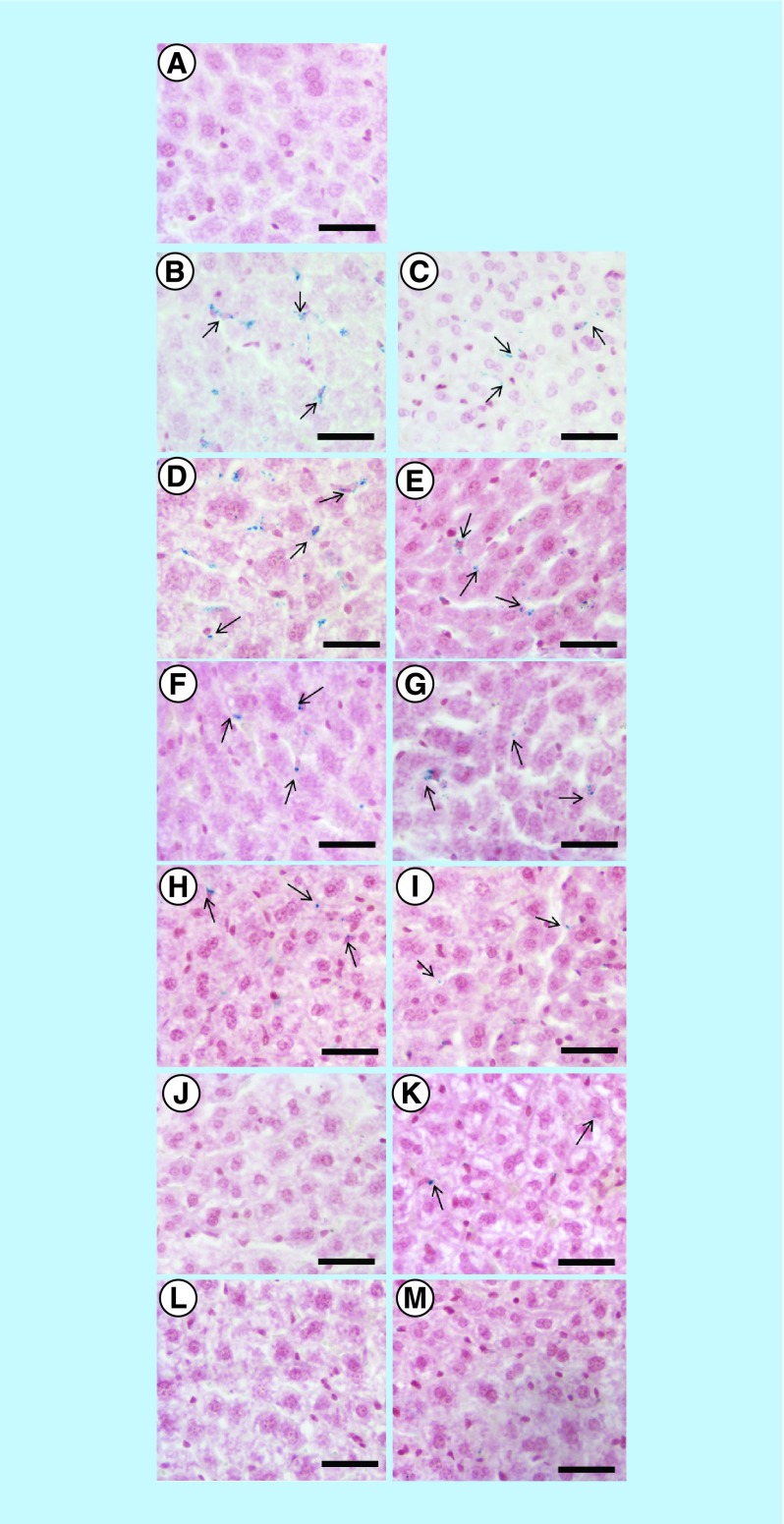
**Qualitative iron detection.** Prussian blue assay in mouse liver of non-injected mouse **(A)** and after 2 h **(B, C)**, 1 day **(D, E)**, 7 days **(F, G)**, 15 days **(H, I)**, 30 days **(J, K)** and 60 days **(L, M)** of Endorem^®^ (left panel) and bioferrofluids (right panel) injection. Black arrows are pointing ferric ions which appear as blue colored dots. Scale bar 50 μm.

Prussian blue assays were carried out to detect free ferric ions in liver and kidney. Results showed that there is an accumulation of both bioferrofluids and Endorem in liver after 2 h of injection, which is indicated by the presence of blue dots in the tissue samples, as shown in Figure 8B & C.

In the case of Endorem, a high amount of iron was detected until 7 days after injection, as shown in Figure 8D & F, and then the amount decreased after 15 days, as shown in Figure 8H. Endorem was not detected after 30 days and above, as shown in Figure 8J & L. In the case of bioferrofluids, although the amount of iron seems to be less to that of Endorem, it exists for a longer period. Iron was detected until 30 days after bioferrofluids injection, as shown in Figure 8E, G, I & K. Bioferrofluids were not detected after 60 days of injection, as shown in Figure 8M. Table 1 shows a perspective idea regarding ferric ion concentration in liver tissue after CA injection. Prussian blue results confirmed the results obtained by MRI.

**Table 1.** **A perspective idea about ferric ion concentration in liver of mice after contrast agent injection using Prussian blue assay.**

**Time**	**Endorem^®^**	**Bioferrofluids**
0	-	-

2 h	+++	+

1 day	+++	++

7 days	+++	+

15 days	+	+

30 days	-	+

60 days	-	-

In the case of kidney, an accumulation of ferric ions was just detected in the period between 2 h and 15 days after Endorem injection. However, in the case of bioferrofluids, ferric ions accumulate until 60 days after injection (Supplementary Figure 6).

### *In vivo* toxicity studies

**Figure 9.**
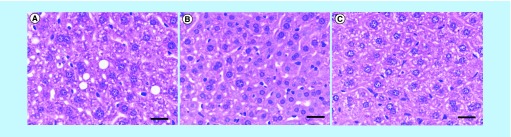
***In vivo* toxicity study.** Hematoxin and Eosin stain in liver of non-injected mouse **(A)** and after 7 days of bioferrofluids **(B)** and Endorem^®^ **(C)** injection. Scale bar 25μm.

Both Endorem and bioferrofluids did not generate any notable histological lesions in liver at different time points (2 h and 1, 7, 15, 30 and 60 days) after CA injection. Figure 9 is an example of the histopathological studies in mouse liver after 7 days of CA injection. A thorough study on the bioferrofluids toxicity and their biodistribution is in preparation and will be published elsewhere.

## Discussion

Obtained results show that both bioferrofluids and commercial Endorem exhibit similar behavior in shortening T_2_ relaxation time, since an increase in the nanoparticles concentration is associated with a decrease in SI, as shown in Supplementary Figure 1. The efficiency of T_2_ CAs depends on r_2_ more than on r_1_. It has been reported that the higher the r_2_/r_1_ ratio the better the efficiency of a negative CA [31,32]. r_1_ and r_2_ relaxivities are dependent on the nanoparticle size in a direct proportion relationship. The effect of nanoparticle size on the r_1_ and r_2_ relaxivities of the here studied bioferrofluids as compared with Endorem has been investigated by Amiri *et al*. [27]. Therefore, it was expected that r_1_ and r_2_ values for bioferrofluids (D_p_ = 13 nm) were higher than for Endorem (D_p_ = 6–9 nm). On the contrary, bioferrofluids show lower values of r_2_ and r_1_ as compared with Endorem. Amiri's results show that the r_2_ value in bioferrofluids of D_p_ = 15 nm is higher than in the case of Endorem. Differences between Amiri's and our study are in the operating frequencies. The experiments here reported were carried out at a frequency of 200 MHz (corresponding to 4.7 T clinical imager). Instead, Amiri *et al*. carried out their experiments at lower frequencies (8.5, 21 and 63 MHz corresponding to about 0.2, 0.5 and 1.5 T clinical imagers, respectively). In their study it is evident that r_1_ is size dependent at low frequencies (0.01–0.1 MHz); larger nanoparticle sizes have higher r_1_ values. At higher frequencies (10–100 MHz), a decrease in r_1_ values was reported for all bioferrofluid samples, Endorem showing higher r_1_ comparative values. This may explain the lower value of r_1_ obtained by our bioferrofluids as compared with Endorem at 200 MHz. On the other hand, r_2_ was found to be frequency independent in the frequency range between 4 and 80 MHz, with values corresponding to Endorem being somewhat lower than the corresponding ones for particles of 15 nm and somewhat higher than those for particles of 10 nm. It seems that this behavior extends to higher frequencies given the slightly lower values of r_2_ obtained by our bioferrofluids as compared with Endorem at high frequency (200 MHz). The efficiency of a T_2_ contrast depends on its r_2_ relaxivity as well as the r_2_/r_1_ ratio: the higher the ratio of r_2_/r_1_, the better the efficiency of a T_2_ CA [31,33]. The calculation of the r_2_/r_1_ ratio for both bioferrofluids and Endorem shows that our bioferrofluids have a higher r_2_/r_1_ ratio than Endorem and this could be an advantage of our bioferrofluid compared with commercial Endorem.

The bioferrofluids and Endorem efficiency as MRI CAs in steady-state condition was evaluated by acquiring T_2_*-weighted images in brain before (pre-contrast) and during 2h post-contrast injection. While 2 h after injection of Endorem the brain vasculature was still hypointense, see Figure 5C, in the case of the bioferrofluids R had recovered to pre-injection values (Figure 5G & Supplementary Figure 2). This indicates a complete clearance of bioferrofluids from the circulation at this time point. It is clear that the bioferrofluids have a half-life time in blood shorter than Endorem. The fast clearance of the nanoparticles from the circulation could be by reticuloendothelial system ‘opsonization’. Opsonization depends on several factors, such as nanoparticle size, charge, surface coating and dose. Coating the nanoparticles with a hydrophilic polymer that reduces the non-specific binding of opsonin proteins could reduce the opsonization effect. Thus, the magnetite core of Endorem is coated with dextran while the bioferrofluids here studied are composed of maghemite cores coated with P4VP-g-PEGA. Several studies show that the long blood circulation time is achieved by increasing the chain length of PEG [19–22]. The surface of the nanoparticles in the P4VP-g-PEGA bioferrofluids is formed by 1/10 in weight of PEGA long chains (MW = 1000) and 9/10 of PEGA short chains (MW = 200). Therefore, these short chains of PEG might not protect the nanoparticles from non-specific protein adsorption and subsequent opsonization, leading to short blood circulation time. A study to identify protein corona formation is recommended. In addition to the surface coating, the nanoparticle size plays a crucial role in nanoparticle opsonization; larger size nanoparticles are cleared faster than smaller size ones. The hydrodynamic diameter of bioferrofluids is 163 nm, while for Endorem is 110 nm. Therefore, the large size of bioferrofluids increases their chance for opsonization and clearance from the circulation. Regarding rCBV maps, Endorem seems to contrast well the space arrangement of cerebral blood vessels, while the bioferrofluids show a darker image (Figure 5D & H).

The dosage of iron administered in brain imaging in the present study may appear considerably high in comparison with the clinical dosage of Endorem. It is to be considered that the dose of CA injected strongly depends on the specific application. Iron-oxide nanoparticles are used in brain studies because they remain confined for long time intervals in the vascular space, differently from small molecular weight CAs (e.g., Gd-DTPA) that are rapidly cleared from blood. This property allows measurement of rCBV and its alterations due to pathologies or to stimulations. High dosages of iron-oxide are administered in brain CBV studies to create a large contrast between pre- and post-contrast images. In a recent review, Kim *et al*. [34]. advise 5–15 mgFe/kg to measure CBV. Wu *et al*. [35] measured microvascular volume distribution in mice using a 30 mgFe/kg injection at 9.4 T and demonstrated that CBV in the cerebral cortex is higher in transgenic APP mice, an experimental model of Alzheimer's disease, than in controls. CBV-weighted functional MRI (fMRI) has been extensively used in experimental fMRI [36,37]; thanks to the increased sensitivity of CBV-weighted contrast compared with classical blood oxygen level-dependent (BOLD) contrast. At 7 T, 15-20 mg Fe/kg ultrasmall superparamagnetic iron oxides improve functional contrast-to-noise ratio (CNR) by 70% in rats compared with BOLD contrast [38]. Mandeville *et al*. [39] reported that CNR in CBV-weighted fMRI with 28 mgFe/kg improved twofold compared with BOLD upon pharmacological stimulation with cocaine. Even in humans, relatively high dosages of ultrasmall superparamagnetic iron oxides of 7 mgFe/kg have been used in fMRI acquisitions [40].

MRI studies related to the accumulation of both CAs in liver 2 h after injection at a dose of 20 μmol Fe/kg body weight indicated no significant difference between bioferrofluids and Endorem. Estimation for the iron concentration in liver after 2 h post-contrast injection yields a similar concentration of 0.05 μM for both CAs. The similarity between both CAs in the estimated accumulated iron concentration in liver tissues is consequent with their similarity in decreasing the T_2_ and T_2_* values. Qualitative detection of iron using Prussian blue assay shows a higher amount of iron in the liver of mice injected with Endorem than with bioferrofluids, as shown in Figure 8B & C. There may be two explanations supporting these results. First, Prussian blue is not a sensitive method; however, it confers optical perspective idea about the existence of ferric ion in liver. A second explanation can be the presence of Endorem^®^ in Kupffer cells as agglomerates. However, our bioferrofluids are distributed in Kupffer cells and hepatocytes.

The presence of CAs in liver was evaluated at several time points until 60 days post-injection. By increasing the time a decrease in darkness of the liver was observed (Supplementary Figure 4), which indicates CA degradation and clearance from liver tissue. Both T_2_ and R values show time dependence. T_2_ values between bioferrofluids and control (pre) show a significant difference until 30 days after injection, while R values show significant difference until 15 days after injection. In the case of Endorem, both T_2_ and R values show statistically significant difference to the control until 15 days after injection. These results are confirmed by Prussian blue assay. Bioferrofluids are observed as blue colored dots until 30 days post-injection (Figure 8K) and disappear at 60 days after injection (Figure 8M), while Endorem is observed until 15 days after injection (Figure 8H) and absent at 30 and 60 days after injection (Figure 8J & L). These results indicate that our bioferrofluids persist for a longer time than Endorem in liver with no toxic effects observed in mice.

Prussian blue assay shows only few blue colored dots in kidney of mice injected with Endorem and bioferrofluids, as shown in Supplementary Figure 6. Detected iron by Prussian blue does not confirm CAs accumulation in kidney, as the source of iron in kidney (exogenous or endogenous) is unknown.

Bioferrofluids have advantages and disadvantages compared with Endorem. In brain studies, bioferrofluids demonstrated less efficacy in decreasing brain signal and faster clearance from blood so Endorem is superior for brain imaging in both first passage and steady state techniques. In liver imaging, the two CAs produced similar efficacy, but bioferrofluids remained in liver for longer time intervals. The long-time permanence of CA in the liver can be an advantage in the clinical settings allowing first the diagnosis of a liver pathology and then monitoring after treatment without the need for a second injection [41]. Finally, bioferrofluids have transversal relaxivity smaller or at most equal to Endorem but higher r_2_/r_1_ ratio that contributes to the efficiency of a T_2_ relaxing CA.

## Conclusion

Our bioferrofluid is a good T_2_ CA with a higher r_2_/r_1_ ratio than commercial Endorem. It has shorter blood circulation time compared with Endorem and they are efficient reticuloendothelial system agents as their persistence is observed in liver tissue. Our bioferrofluid persists in liver for longer period of time (up to 30 days post-injection) than Endorem with no toxic effect observed in liver tissue. Moreover, our bioferrofluid has shown a much higher magnetic heating power than Endorem [10], so it would be valuable for theranostics. Accumulation of CAs in kidney was not clear and requires further studies to be confirmed. Studies pertaining to protein adsorption on the nanoparticle surface ‘protein corona’ are recommended.

## Future perspective

SPIONs are an attractive platform for biomedical applications. In recent years, we have been developing a multifunctional nanoplatform of polymer-based SPIONs, which can be functionalized with antibodies, drugs, etc. Several studies were carried out to study the *in vitro* toxicity, *ex vivo* hematotoxicity, *in vitro* relaxivity and *in vitro* magnetothermic responses. Obtained results from these studies make these nanoparticles as good candidates for theranostics. The present study represents a complementary part of the previous studies. Here, we investigate the diagnostic potential of our nanoparticles as T_2_ CA for MRI *in vivo*, their uptake and toxicity in liver. Studies of *in vivo* toxicity, biodistribution, biodegradability and excretion will be carried out in the near future as unavoidable studies required before considering access to clinical phases.

**Summary points**

- Obtained results from previous *in vitro* cytotoxicity, *ex vivo* blood compatibility, relaxometry and magnetothermal studies make these bioferrofluids a good candidate for theranostics.
- Bioferrofluids is a good T_2_ contrast agent with a higher r_2_/r_1_ ratio than commercial Endorem^®^.
- Bioferrofluids have shorter blood circulation time in comparison with Endorem.
- Bioferrofluid persists in liver for longer period of time (up to 30 days post-injection) than Endorem.
- Bioferrofluids have no toxic effect in mice liver.
